# Trace eyeblink conditioning is associated with changes in synaptophysin immunoreactivity in the cerebellar interpositus nucleus in guinea pigs

**DOI:** 10.1042/BSR20170335

**Published:** 2018-05-08

**Authors:** Rui Li, Qi Li, Xiao-Lei Chu, Tao Tao, Lan Li, Cheng-Qi He, Fang-You Gao

**Affiliations:** 1 Department of Rehabilitation, Guizhou Provincial People’s Hospital, Zhongshan East Road 83, Guiyang 550002, Guizhou, China; 2 Department of Rehabilitation, Tianjin Hospital, Jiefang South Road 406,Tianjin 300211, China; 3 Department of Clinical Laboratory, Guizhou Provincial People’s Hospital, Zhongshan East Road 83, Guiyang 550002, Guizhou, China; 4 Department of Rehabilitation Medicine, West China Hospital, Sichuan University, No. 37, Guo Xue Xiang, Chengdu 610041, Sichuan, China; 5 Department of Neurosurgery, Guizhou Provincial People’s Hospital, Zhongsan East Road 83, Guiyang 550001, Guizhou, China

**Keywords:** interpositus nucleus, learning and memory, synaptic plasticity, synaptophysin, trace eyeblink conditioning

## Abstract

Synaptic plasticity plays a role during trace eyeblink conditioning (TEBC). Synaptophysin (Syn) is a major integral transmembrane protein, located particularly in the synaptic vesicles, and is considered a molecular marker of synapses. In addition, Syn immunoreactivity is an important indicator of synaptic plasticity. In the present study, we used immunohistochemical techniques to assess changes in Syn expression in the cerebellar interpositus nucleus (IN) of guinea pigs exposed to TEBC and pseudoconditioning. Additionally, we analyzed the relationship between Syn immunoreactivity and the percentage of trace-conditioned responses. Guinea pigs underwent trace conditioning or pseudoconditioning. Following two, six, or ten sessions, they were perfused and the cerebellum was removed for Syn immunohistochemical evaluation. After sessions 6 and 10, a significant increase in conditioned response (CR) percentage was observed in the trace-conditioned group, with the CR percentage reaching the learning criteria following session 10. Besides, for trace-conditioned animals, the Syn expression in IN was found significantly up-regulated after session 10 compared with pseudoconditioned ones. Our data suggest that the increase in Syn expression links to synaptic plasticity changes in the cerebellar IN and provides a histological substrate in the IN relating to TEBC training. The changing trend of Syn immunoreactivity in the IN is associated with CR percentage.

## Introduction

Classical eyeblink conditioning (EBC) has been widely used in studies on behavioral and neurobiological mechanisms of associative learning and memory [1,2]. Many studies suggest that the cerebellum is a necessary neural structure for the establishment of delayed EBC (DEBC) [3–5]. Lesion experiments show that the establishment and expression of EBC depend on the integrity of the cerebellar interpositus nucleus (IN) [6,7]. Additionally, electroneurophysiology data showed that cerebellar IN neurones had significant changes in electrodischarge activity relating to learning during the establishment of EBC [8]. Studies using functional imaging of brain activity also found that the cerebellar IN was activated during the establishment of EBC [9].

Previous studies have shown that the cerebellar IN, not the hippocampus, is necessary for early acquisition, short-term, and long-term retention of DEBC [10,11]. It is believed that the hippocampus is essential for the initial learning and retention, but not long-term retention of DEBC, and that the effect of the cerebellum is not important [12]. DEBC develops when a sound or light is presented as a conditioned stimulus (CS) and a corneal airpuff or periorbital electric shock is presented as an unconditioned stimulus (US); the CS comes first, followed by the US and both are terminated at the same time. Trace EBC (TEBC) has some similarity to TEBC. But in TEBC, an additional interstimulus interval without any stimuli (trace interval) exists between the CS and US. Both types of EBCs are dependent on the cerebellum–brainstem circuit; however, TEBC requires additional modulations from structures outside the cerebellum.

Learning and memory are advanced functions of the brain. Learning occurs when the central nervous system recognizes changes in the surroundings and subsequently obtains new behavioral habits, and memory is used to store and maintain these experiences after learning. In recent years, a number of studies have observed changes in synaptic morphology and functional plasticity following the establishment of learning and memory [13]. The postsynaptic density is the specific structure on the inner side of the postsynaptic membrane that participates in the regulation and integration of postsynaptic signal transduction, and plays an important role in the formation of learning and memory [14,15]. Previously, we observed that in an EBC paired training group, the postsynaptic density of the cerebellum IN was thickened and its synaptic activity regions were extended, suggesting the establishment of EBC is related to changes in the ultrastructure of the postsynaptic membrane of IN neurones [16]. However, few studies have reported synaptophysin (Syn) changes in IN neurones following TEBC training. Since IN neurones are involved in TEBC, the amount of Syn in IN neurones may change following TEBC training.

Some studies suggest that Syn plays a crucial role in synaptic plasticity through regulating the endocytosis and exocytosis of neurotransmitters [17–19], the formation/stabilization of synapses, and synaptic transmission efficiency [20]. It is also known that Syn plays a role in the formation and recycling of synaptic vesicles; learning and memory are closely related to Syn immunoreactivity. Thus, Syn provides a molecular marker of synapses, and the level of Syn immunoreactivity could be an important indicator of synaptic plasticity. However, so far, it has not been reported whether plasticity changes in the presynaptic membrane are involved in EBC. It has been observed that a decrease in the expression of Syn-positive products is related to impaired learning and memory ability [21]. Therefore, using the TEBC model established in guinea pigs, the present study aims to explore any changes in presynaptic Syn levels in cerebellar IN neurones, and investigate the presynaptic mechanisms underlying the establishment and expression of EBC.

## Materials and methods

### Subjects

Twenty-four adult male albino Dunkin–Hartley guinea pigs weighing between 600 and 700 g (5–6 months old) were randomly assigned to two groups: a trace-conditioning group (*n*=4 for each time point) and a pseudoconditioning group (*n*=4 for each time point). Before the experiment and between conditioning sessions, the animals were individually housed in standard plastic cages on a 12/12 light/dark cycle with free access to food and water. The room temperature was maintained at 25 ± 1°C. All experiments were performed between 8:00 a.m. and 6:00 p.m. The experimental procedures were approved by the Animal Care Committee of the Guizhou Provincial People’s Hospital and were performed in accordance with the principles outlined in the NIH Guide for the Care and Use of Laboratory Animals. All efforts were made to minimize the use of animals and, where use was necessary, to optimize comfort.

### Surgery and apparatus

Surgical procedures were performed in an aseptic environment using sterilized surgical tools. Guinea pigs were anesthetized and their heads were fixed in stereotaxic apparatus (SR-6N; Narishige, Tokyo, Japan). A Plexiglas headstage (1.0 cm × 1.0 cm × 0.5 cm), designed to secure the animal’s head and to hold the airpuff pipe, was attached to anchoring screws using dental cement on the skull surface. A small nylon loop was sutured into the edge of the left upper eyelid. In the present study, this loop was utilized to attach the left upper eyelid to a movement-measuring device. After the surgery, guinea pigs were allowed 1 week of recovery. Post surgery, all guinea pigs gained weight normally and showed no abnormal behavior.

### Behavioral training procedures

Following postoperative recovery, guinea pigs were restrained in a square Plexiglas container located in a ventilated sound- and light-attenuating chamber and were adapted to the experimental environment for 2 days (90 min/day). During environmental adaptation, the animals’ heads were secured with blunt ear bars pressing on the headstage, but no training stimuli presented. The left eye of the animal was held open, with the nylon loop linked to the swivel arm of a high-resolution spring return potentiometer. Moreover, the animal’s left lower eyelid was taped open. These two measures were made to ensure continual exposure of the left cornea. Following environmental habituation, the animals were randomly assigned into two groups for daily sessions of training.

For the trace-conditioning group, the CS was presented paired with the US, but with a 150-ms interval separating CS offset and US onset. A speaker located above and behind the animal at a distance of 10 cm delivered the CS tone (1 kHz, 80 dB, 300 ms, 5 ms rise/fall time). An airpuff tube was placed approximately 1 cm from the animal’s left eyeball and served to deliver the airpuff US (3 psi, 200 ms). The airpuff was created by the release of compressed air and its intensity was adequate to evoke an eyeblink. The daily trace-conditioning training session consisted of 100 CS-US trials with a 30 ± 10 s (mean =30 s) interval, and lasted for 90 min. The pseudoconditioning training session also consisted of 100 trials; however, for each trial, the CS was presented unpaired in relation to the US.

The acquisition of TEBC required ten sessions; animals did not undergo any training in sessions 11–14 but were trained again in session 15 using the same parameters used during acquisition training. Training occurred on successive days during the acquisition of TEBC, until the animal was selected for immunohistochemical processing.

### Immunohistochemistry

**Figure 1:**
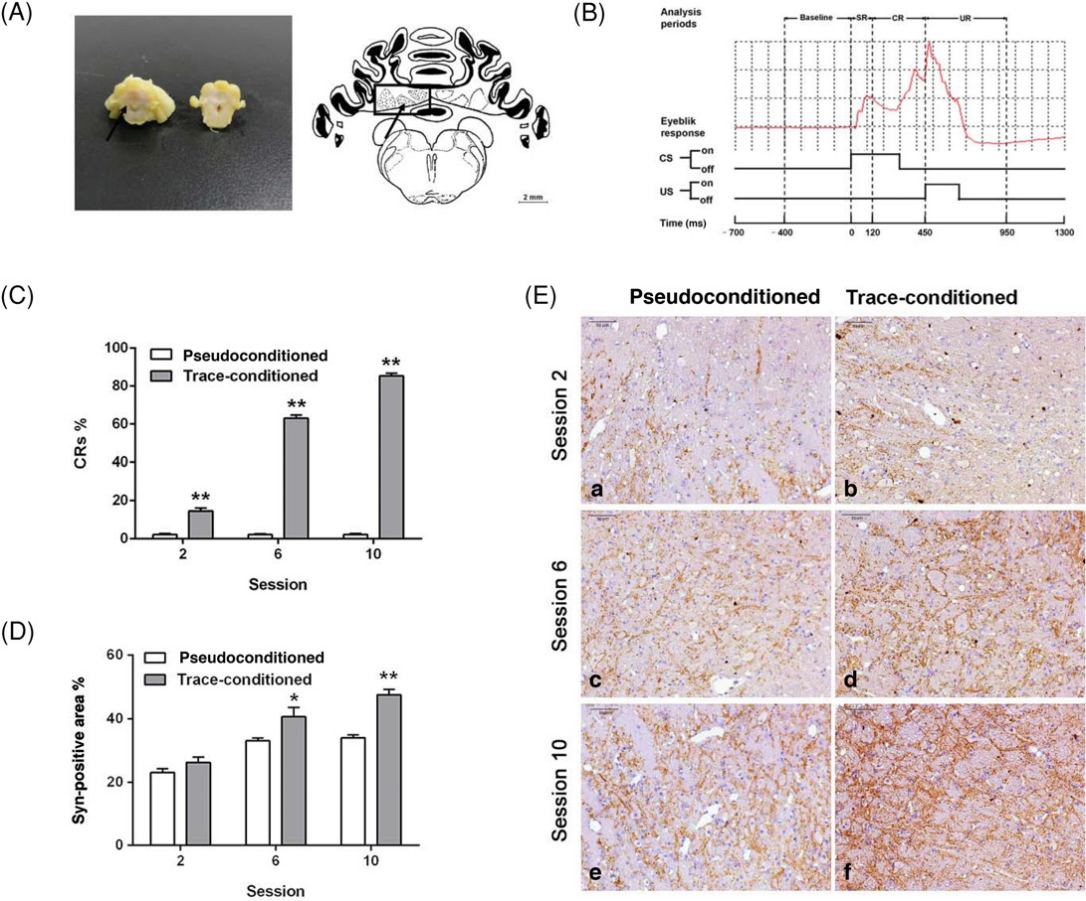
TEBC is associated with changes in Syn immunoreactivity in the cerebellar IN (**A**) Location of guinea pig cerebellar IN. Left: a representative sample of the coronal cutting surface of the guinea pig cerebellum; the black arrow indicates the location of the cerebellar IN. Right: a schematic drawing of the coronal section; the location of the cerebellar IN is indicated by the black arrowhead. (**B**) CS, US, and analysis periods during TEBC. In a CS-US paired trial, we analyzed the parameters of SR (1–120 ms period after the CS onset), eyeblink CR (121–450 ms period after the CS onset), and UR (1–500 ms period after the US onset). These responses were based on the average amplitude of baseline (1–400 ms period prior to the onset of the CS). An example of a typical SR, CR, and UR is illustrated by the red curve. (**C**) Percentage of CRs in sessions 2, 6, and 10. The trace-conditioned group (*n*=4) showed a large increase in CR percentage. (**D**) Syn immunoreactivity changes in the cerebellar IN following the second (a,b), sixth (c,d), and tenth (e,f) sessions. (a), (c), and (e) represent the pseudoconditioned trace-conditioned group (*n*=4); (b), (d), and (f) represent the trace-conditioned group (*n*=4). (**E**) Syn immunoreactivity staining in the cerebellar IN following the second, sixth, and tenth session. The trace-conditioned group shows an increase in Syn expression with time; and at sessions 6 and 10, the Syn expression was significantly higher in the trace-conditioned group than in the pseudoconditioned group. *P<0.05; **P<0.01.

The cerebellum (Fig. 1A) was dissected and fixed in 4% paraformaldehyde (PFA) for 24 h at 4°C. Tissues were processed for paraffin embedding and sagittal sections (5-μm thick) were collected. In brief, after blocking endogenous peroxidase activity, increasing permeability (0.5% Triton X-10, 30 min), and blocking (3% BSA, 1 h at 37°C) sections were exposed to the anti-Syn antibody in 1% BSA (12 h, 4°C, 1:200; Merck, Darmstadt, Germany). Sections were then washed by PBS for three times and incubated with the avidin–biotin complex (DAKO, Glostrup, Denmark) followed by the biotinylated secondary antibody (1:200; 2 h, 37°C; DAKO, Glostrup, Denmark), and finally staining was visualized using the 3,3-diaminobenzidine substrate kit (Vector Laboratories, Burlingame, CA, U.S.A.). Finally, sections were observed under a microscope (Leica DM2500, Germany).

### Behavioral data collection and analysis

For conditioning (Fig. 1B), a 2000 ms period was recorded during each trial, starting 700 ms before the onset of the CS. All data presented are measurements of left upper eyelid movements. The parameters of eyeblink responses were analyzed offline using Matlab software (v. 6.5). For each CS-US paired trial, we calculated the average above-threshold amplitude value for the baseline (BL; 1–400 ms before the CS onset), startle eyeblink response (SR; 1–120 ms after the CS onset), conditioned response (CR; 120–450 ms after the CS onset), and unconditioned eyeblink response (UR; 1–500 ms after the US onset). A significant eyelid movement was defined as an increase in integrated activity that was greater than mean baseline amplitude plus four times the S.D. of the baseline activity. For each CS-alone trial in the pseudoconditioned group, a significant CR occurring in the period of 121–450 ms after the CS onset was defined as a CR-like eyeblink response.

### Syn immunohistochemistry collection and analysis

Photographs were collected with a full automatic image analysis system (Germany KONTRON IBAS2.O, JVC ky-F30B 3-CCD color video image input apparatus) after Syn immunohistochemical processing. The HPIAS-1000 image analysis system was used to measure the optical density value (ODV) of Syn immunoreactants in the cerebellum IN. Simultaneously, ODV of the background zone of each corresponding section was measured in order to calculate a correcting ODV (CODV) by subtracting the background ODV from Syn immunoreactants’ ODV values. CODV was used for analysis and comparison in order to avoid errors caused by non-specific staining. CODV determination was carried out under the same optical conditions.

### Statistical analysis

All data are expressed as the mean ± S.E.M. Statistical significance was determined via Student’s *t* test. The statistical significance level was set as *P*<0.05.

## Results

### Increased CR percentage following trace-conditioning training

The trace-conditioning training produced a significant increase in CR percentage (Figure 1C), with approximately 65% (64.17 ± 4.75%) CRs. However, the pseudoconditioning training group exhibited no significant changes, even a slight decrease in CR percentage. Using the Shapiro–Wilk normality test, all the data were shown to be normally distributed (trace-conditioned group; session 6, w =0.831, *P*=0.109; session 10, w =0.941, *P*=0.671). CR percentage in each session was significantly higher in the trace-conditioned group compared with the pseudoconditioned group (multiple Student’s *t* test, *P*<0.01 at sessions 2, 6, and 10).

### Increased Syn expression induced by trace conditioning

In the trace-conditioned group, Syn-positive granules and fiber bundles increased with TEBC training. Prominent formation of Syn-positive granules and fiber bundles could be observed at session 6 (Figure 1D-E). Repeated *t* test indicated a significant effect of session on Syn expression (F (2, 18) =49.6, *P*<0.05) and that the Syn expression at session 6 or 10 was significantly higher than that in session 2 (session 6 compared with session 2, *P*<0.01; session 10 compared with session 2, *P*<0.01). Simultaneously, a significant effect of group (F (1, 18) =34.4, *P*<0.01) on Syn expression was found. The effect of trace-conditioning training on the level of Syn immunoreactivity was significantly higher than that of the pseudoconditioning training at sessions 6 (*P*<0.05) and 10 (*P*<0.01), indicating a link between Syn expression in the IN and trace conditioning.

## Discussion

This research established classical TEBC by conducting behavioral training in guinea pigs, and observing the corresponding changes of Syn expression in the cerebellum. The results showed that CR acquisition in the trace-conditioning group had significantly increased by the sixth session, and reached a peak following the tenth session of behavioral training. Significant differences were observed between the trace-conditioning and pseudoconditioning groups. When TEBC was established, the CODV values of Syn staining in the IN gradually increased with the percentage of CR acquisition. Our results further support that the cerebellar IN is an important part of the nerve loop in the establishment and expression of TEBC, and identify some of the presynaptic mechanisms of cerebellar regulation of learning and memory.

Numerous studies have shown that learning and memory are closely related to Syn immunoreactivity. Some studies show that the expression of Syn in the guinea pig hippocampal CA3 region increases with age [22,23]. For patients suffering from Alzheimer’s disease (AD), mainly characterized by the pathological absence of neurones, the expression of Syn in the hippocampus is significantly lower than in healthy controls [24]. In addition, the density of immune reactants of Syn in the molecular layer of cerebellar lobules I and V decreases with age [25–27]. Rehabilitation training that improves dyskinesia can also counteract the decreasing expression of Syn in cells surrounding lesions and the contralateral motor cortex, caused by the brain injury. Functional electrical stimulation can up-regulate the expression of Syn and simultaneously improve the memory and cognitive ability of AD patients [28–30].

In TEBC training, repeated presentation of the CS paired with the US had a number of effects; it increased the response of the synapse in the cerebellar IN to specific stimuli, enhanced transmission, and enabled the synapse configuration to generate plasticity changes. In the trace-conditioning group, the CR acquisition rate significantly increased with increased training time, and a comparatively stable acquisition rate can be maintained for a certain time. Behavioral training excited cerebellar internal neurones through excitatory afferent fibers (climbing fibers and mossy fibers), leading to axonal sprouting and collateral formation to construct new synapses. As a result, Syn-positive granules increased, reflecting that the number of presynaptic vesicles might be increased. Some researchers have also shown that training in different ways is helpful to increase *Syn* mRNA levels, thereby promoting the formation of synapses.

The present research suggests that increased Syn in the cerebellar IN, as a result of training or direct stimulation, has the potential to become a new therapeutic approach to improve motor-learning impairments. Long-term depression (LTD) in parallel to fiber-cerebellar cortical Purkinje neurone synapses, and the subsequent facilitation effect in PF-IN neuronal synaptic transmission are forms of synaptic plasticity-associated learning [31]. It is generally agreed that the hippocampus plays an important role in the establishment of TEBC. On the contrary, the present study confirms that the cerebellar cortex and deep cerebellar nuclei are both vital in the establishment of TEBC [32], but there still exist controversies for the role of the deep cerebellar nuclei in the establishment and maintenance of TEBC. In addition, we found that Syn levels in the cerebellar IN increased gradually with the acquisition of behavior during the establishment and maintenance of TEBC. Increases in Syn levels represent changes in synaptic structure, supporting the idea that synaptic plasticity in the cerebellar IN may be the structural basis of establishing TEBC. In addition, other researchers have found that the cerebellar IN and cerebellar cortex receive information about the US and CS simultaneously, and it is likely that both structures participate in the integration between the CS and US. Therefore, the changes in synaptic plasticity in the cerebellar IN and cortex in the cerebellar loop need to be further studied.

## Abbreviations

AD: Alzheimer’s disease
CA3: interpositus nucleus
CODV: correcting optical density value
CR: conditioned response
CS: conditioned stimulus
DEBC: delayed eyeblink conditioning
EBC: eyeblink conditioning
IN: interpositus nucleus
ODV: optical density value
PF-IN: parafascicular nucleus-interpositus nucleus
Syn: synaptophysin
TEBC: trace EBC
US: unconditioned stimulus
